# The Pivotal Role of Social Support, Self-Compassion and Self-Care in Predicting Physical and Mental Health Among Mothers of Young Children

**DOI:** 10.3390/healthcare13151889

**Published:** 2025-08-01

**Authors:** Shiran Bord, Liron Inchi, Yuval Paldi, Ravit Baruch, Miriam Schwartz Shpiro, Shani Ronen, Limor Eizenberg, Ilana Gens, Maya Yaari

**Affiliations:** 1 Department of Health Systems Management, The Max Stern Yezreel Valley College, Emek Yezreel 1930600, Israel; lironi@yvc.ac.il; 2 Tovanot Research Institute, Gedera 7044625, Israel; yuval@tovanot.com; 3 Public Health Directorate, Israel Ministry of Health, Jerusalem 9101002, Israel; ravit.baruch@moh.gov.il (R.B.); ilana.gens2@moh.gov.il (I.G.); 4 Goshen Child Health and Wellbeing, Jerusalem 9765418, Israel; miriamss@goshen.org.il (M.S.S.); shanir@goshen.org.il (S.R.); mayay@goshen.org.il (M.Y.); 5 Nursing Division, Israel Ministry of Health, Jerusalem 9101002, Israel; limor.eizenberg@moh.gov.il

**Keywords:** well-being, social support, self-compassion, self-care, motherhood, health promotion

## Abstract

Background: Mothers’ health significantly affects their well-being and that of their families. The early years of motherhood can be tough and impact mental health. This study examined the associations between mothers’ self-compassion, social support, and self-care behaviors and their physical and mental well-being. Methods: In August 2023, an online cross-sectional survey was conducted among 514 Israeli mothers with children under three. Mothers’ physical and mental health was assessed using SF12. Self-compassion was measured by the Self-Compassion Scale (SCS). Social support was evaluated through the Multidimensional Scale of Perceived Social Support (MSPSS), and self-care was assessed via the Pittsburgh Enjoyable Activities Test (PEAT). Results: Respondents’ average age was 31.5 years. Their self-reported physical health was relatively high, with a mean of 78.36 (SD = 21) on a 0–100 scale (n = 442). Mental health scores were lower, with a mean of 65.88 (SD = 20.28, n = 401). Perceived physical health was higher among Jewish mothers, younger mothers, and those with higher income levels. Additionally, greater social support and self-compassion correlated with better perceived physical health (Adj R^2^ = 0.11, *p* < 0.001). For mental health, higher scores were observed among Jewish mothers, younger mothers, and full-time employed mothers. Furthermore, higher social support, self-compassion, and self-care practices were associated with improved perceptions of mental health (Adj R^2^ = 0.39, *p* < 0.001). Conclusions: Promoting the well-being of mothers is crucial for their health, their children’s well-being, and the family unit. Health professionals working with mothers of young children should emphasize and help promote social support, self-compassion, and self-care activities.

## 1. Introduction

After giving birth, women may face a range of physical, emotional and social challenges [1]. They often experience pain and require time for physical recovery. Chronic exhaustion can occur due to the intensive care and supervision that a newborn demands. Emotional difficulties may arise from feelings of social isolation, as women tend to spend much of their time at home with their babies. In recent years, maternal mental health has garnered significant research attention during and surrounding pregnancy, primarily focusing on women experiencing depressive symptoms [2,3,4]. While this period is very important, it is also crucial to study the physical and mental health of mothers with young children (not only directly postpartum), and the factors that may contribute to better health outcomes for them. The current study focuses on studying the physical and mental health of women with young children (up to three years old), emphasizing their daily functioning, well-being, and the factors contributing to their overall health. 

Women’s socio-demographic characteristics may play a significant role in their physical and mental health status. For example, age at birth is an important factor in predicting physical and mental health among mothers of young children. A national study in the USA found high levels of current health problems among women who first gave birth during or shortly after puberty. Problems drop steadily the longer that first birth is delayed, up to about age 34, then rise increasingly steeply, particularly after about age 40 [5]. In addition, very young women may face unique challenges, such as inexperience or insufficient support, while older women may experience various health and emotional stresses. Socioeconomic status also affects the mother’s health status. A study conducted by the American Psychological Association found that new mothers who feel that they are low on the socioeconomic scale suffer from more health problems a year after giving birth than mothers who feel that they are of a higher status [6]. Another relevant issue is ethnicity, with ethnic minority mothers at increased risk for physical and mental complications postpartum. For example, research showed that African American and Hispanic women tend to experience physical health complications after childbirth at higher rates than white women, including high blood pressure and diabetes [7]. Ethnic minority mothers are also at higher risk of psychological distress postpartum, while they are less likely to be treated for anxiety or depression [8]. 

Differences in level of religiosity can affect the experience of adapting to a new baby. These may stem from community support, social values and/or approaches to family life and motherhood. Studies indicate that religious women tend to enjoy more community and family support than secular women, which helps them cope better with the challenges of motherhood. This support includes not only physical help, but also significant emotional support, which can reduce the pressures and difficulties associated with motherhood [9]. This may be defined as social support, which relates to the interpersonal exchange between two or more people in an effort to enhance the recipient’s well-being. Social support might be actually received or subjectively perceived [10,11,12]. Research shows that social support provided appropriately to mothers can reduce stress and improve their well-being [13]. 

The concept of self-compassion, originally Buddhist, has been integrated into Western psychological literature [14]. Neff [15] describes self-compassion as being supportive toward oneself when experiencing suffering or pain, whether caused by personal mistakes, inadequacies or external life challenges. The multifaceted construct of self-compassion is theorized as a bipolar continuum, ranging from an uncompassionate to a compassionate self-response [15]. This construct encompasses three main components: extending kindness and understanding to oneself instead of engaging in strict self-criticism; viewing one’s experiences as part of the broader human experience rather than feeling isolated; and practicing mindful awareness instead of over-identifying [16]. Research indicates that mothers with higher self-compassion reported being less influenced by feelings of guilt and hence enjoying higher levels of self-engagement in health-promoting behaviors, such as physical activity, healthy eating and getting enough sleep. Therefore, self-compassion may provide mothers with a constructive way to address any guilt they feel about prioritizing their own health [17] and consequently improve their physical and mental health. Additionally, research indicates that self-compassion can reduce the association between self-criticism and depression among postpartum women [18]. Therefore, self-compassion may help enhance positive well-being and mental health during the perinatal and early postnatal stages. A study examining the relationship between self-compassion and mothers’ well-being revealed that higher levels of self-compassion were significantly associated with lower body shame, anxiety, and depressive symptoms, as well as greater life satisfaction, self-esteem, and positive maternal experiences [19]. Moreover, self-compassion seems to be a strong coping mechanism and source of resilience in the face of life stressors such as divorce [20]. 

Hewitt and her colleagues [21] identified a reciprocal relationship between daily pressures and the mental and physical health of Australian mothers with children aged 0–4. They found a notable negative correlation between time pressure and mothers’ physical health, while a stronger, ongoing relationship and reciprocal influences were observed concerning their mental health. This leads us to the concept of Self-Care, emphasizing the necessity of allocating time for oneself. Measurable enjoyable leisure activities are defined as the enjoyable pursuits individuals choose when free from work and life responsibilities, such as savoring coffee, taking siestas, engaging in social activities, embracing sports, pursuing hobbies and/or relaxing in nature [22]. Studies have shown that individuals who engaged more in enjoyable leisure activities experienced higher levels of positive affect in their daily lives than those who participated less in such activities [23,24]. Individuals who participate in more enjoyable leisure activities function better psychologically and physically, and report a more positive affect, life satisfaction, lower depression and more social support [22]. Enjoyable leisure activities were found particularly beneficial for unemployed people, contributing to their mental health by providing a sense of daily structure and improving their perception of spending time more effectively [25]. 

The current study was designed to learn about the role of social support, self-compassion and self-care in predicting physical and mental health among mothers of young children. As most studies on mothers’ physical and mental health have focused on pregnancy and mothers of first-borns, the current study suggests broadening the scope to include mothers of young children as a group of interest. The significance of this study lies in its focus on the critical early stage of motherhood—a period characterized by profound physical, emotional, and psychological changes. During this sensitive time, many women face health and mental challenges that can impact their and their children’s well-being. This knowledge will enhance the development of tailored and effective interventions aimed at promoting maternal well-being and supporting the well-being of the entire family.

The hypothesis is that higher social support, self-compassion and self-care will be associated with improved physical and mental health among mothers. Further, we hypothesized that we would find differences in the mothers’ physical and mental health based on their socio-demographic characteristics (ethnicity, age, employment status and income level). 

## 2. Materials and Methods

### 2.1. Participants and Procedures

The current study used a cross-sectional design to examine the associations between mothers’ self-compassion, social support, and self-care behaviors and their physical and mental well-being. The data were collected during August 2023 after obtaining all necessary ethical approvals (2023-72 YVC EMEK). Data collection was carried out through a large online panel survey company (IPANEL). The study’s inclusion criteria were women with at least one child aged 3 years or younger. The only exclusion criterion was lack of consent to participate. Participants were 514 mothers with infants and toddlers up to three years old. The mothers were approximately 31.5 years old on average (SD = 5.64) and mostly married. They had two children on average (M = 1.76, SD = 1.08), and their youngest child was 14 months old on average (SD = 9.31). Most mothers had already completed their maternity leave (approximately 78%). Close to 70% of the mothers had an academic degree, approximately three-fourths were employed, and approximately 57% had an average or above-average income. Most mothers were Jewish, secular and urban (Table 1).

### 2.2. Measures

To measure the mother’s physical and mental health, we used the Short Form Health Survey, a comprehensive, reliable yet short and easy-to-use tool to assess mental health, health perceptions, social and role functioning, physical functioning and pain [26]. SF-12 is the shorter version of SF-36 and is used extensively [27]. Responses are given on a six-point Likert scale ranging from not at all (1) to every day (6). In the current study, we used all 12 items to measure the women’s physical and mental health. The total score is the sum of the items in each scale, multiplied in a manner such that the final total score ranges 0–100 (physical health—α = 0.77; mental health—α = 0.74). A higher score represents better perceived health.

Social support was measured by Zimet’s [28] 12-item Multidimensional Scale of Perceived Social Support (MSPSS, α = 0.94), which is short and easy to use and has been used frequently [29,30]. Responses are given on a seven-point Likert scale ranging from very strongly disagree (1) to very strongly agree (7). The total score is the mean of the items, such that a higher score reflects greater social support.

Self-compassion was measured using the Self-Compassion Scale (SCS) based on six components: increased self-kindness, common humanity, mindfulness, reduced self-judgment, isolation and overidentification. Responses are given on a five-point Likert scale ranging from almost never (1) to almost always (5). The total score is the mean of the items, such that a higher score reflects greater self-compassion. Many studies have extensively validated the long and short versions of the SCS [31,32]. This study included 12 items (α = 0.85) out of 18 in the original scale. 

Self-care was measured using the mother’s self-report on enjoyable activities using the Pittsburgh Enjoyable Activities Test questionnaire (PEAT). This assessment tool was developed to measure the frequency with which people participate in enjoyable activities and their impact on their psychological and physical well-being. The questionnaire includes a list of daily activities, and participants are asked to report the frequency of their participation in each activity during the past month (α = 0.84) [22]. Responses are given on a five-point Likert scale ranging from almost never (0) to every day (4). The total score is the sum of the items, such that a higher score reflects involvement with more enjoyable activities.

Socio-demographic and background variables: Income was defined as an ordinal variable and measured using a five-interval scale representing different income levels, with the middle category corresponding to the national average wage. Employment was categorized into two groups: working mothers and non-working mothers. Age was defined as a continuous variable and measured in years. Ethnicity was measured as a dichotomous variable with two categories: Jewish and Arab.

### 2.3. Data Analysis

Data were analyzed with SPSS ver. 29. Descriptive statistics were used for the demographic and socioeconomic characteristics, and internal consistencies for the study variables were examined with Cronbach’s α. Skewness values ranged from –1.34 to 0.14 (SE = 0.11), and kurtosis values ranged from –0.34 to 1.41 (SE = 0.22), indicating normal distributions and allowing the use of parametric statistics. The study variables were described with means and standard deviations, and Pearson correlations were calculated between them. The demographic and socioeconomic associations for the dependent study variables were examined with Pearson correlations and analyses of variance. Multiple regression analyses were calculated for the dependent study variables (physical and mental health) with demographic and socioeconomic characteristics, social support, self-compassion and the implementation of enjoyable activities. As the level of income (a 5-point ordinal variable) did not deviate from a normal distribution (skewness = 0.11, SE = 0.11), it was used as a continuous variable. No collinearity was detected in the regression analyses (highest VIF value = 1.34). The Bonferroni correction for multiple comparisons was applied in bivariate analyses, and in multivariate analyses *p* was set at <0.05.

Sample size was calculated with G*Power 3. For a multivariate analysis of variance with two response variables, and two to three groups, a low effect size of f^2^ = 0.22, α = 0.05, and power = 0.80, the required sample size is N = 432 participants. For a multiple regression analysis with up to ten predictors, a low effect size f^2^ = 0.04 (equals R^2^ = 0.04), α = 0.05, and power = 0.80, the required sample size is N = 416 participants. 

## 3. Results

### 3.1. Descriptive Results

The participating mothers’ physical health was relatively good on average, with 86% of them scoring above 50 (n = 442). Their mental health was somewhat lower, although still relatively good, with 78% of them scoring above 50 (n = 401). Social support was reported as relatively high, and self-compassion was moderate on average (see Table 2). The respondents’ participation in enjoyable activities was moderate-to-low on average. Positive and significant correlations were found among all variables. That is, higher levels of social support, self-compassion and enjoyable activities were associated with better physical and mental health, and social support, self-compassion and enjoyable activities were positively associated amongst themselves.

Several socio-demographic and socio-economic associations were found for the study variables (Table 3). First, Jewish mothers reported higher physical and mental health than Arab mothers. Second, mothers who were full-time employed or part-time employed for more than half of a full-time job, or on maternity leave, reported higher mental health than mothers who were unemployed, housewives or employed for less than half a full-time job. Third, mothers with higher income levels tended to report higher levels of physical health. Other socio-demographic and socio-economic characteristics were not associated with physical and mental health (i.e., age, number of children at home, time from last baby delivery, time since end of maternity leave, level of education, and level of religiosity).

### 3.2. Physical and Mental Health

In order to assess the relative contribution of the demographic and socio-economic characteristics, as well as the study variables, to the mothers’ physical and mental health, two multiple regression analyses were calculated (Table 4). In light of the results shown in Table 3, the demographic and socio-economic variables used were: ethnicity (1: Jewish, 0: Arab), income (scored 1–5 and regarded as continuous), and employment status (1: full time, part time above half of full-time job, and maternity leave, 0: unemployed, housewife, employed less than half of full-time job). Due to the relatively wide range of the mothers’ ages, age was used as another demographic variable in the regression analyses. Level of education, time since ending maternity leave, and level of religiosity were excluded from the regression models because they were not correlated with physical and mental health (see Table 3). The demographic and socio-economic variables were entered in the first step, and the study variables in the second (see Table 4).

The results indicate that 11% of the variance in mothers’ physical health and 39% in their mental health are explained by these models. Physical health tends to be higher among Jewish mothers, younger mothers, and those with higher income levels. Additionally, greater social support and self-compassion are associated with better perceived physical health, with their contributions being similar (t(513) = 0.23, *p* = 0.818). For mental health, it is higher among Jewish mothers, younger mothers, and women working full-time. Moreover, increased social support, self-compassion, and engaging in enjoyable activities are associated with improved perceived mental health. The contribution of self-compassion to perceived mental health was the highest, greater than that of both social support (t(513) = 2.27, *p* = 0.024) and enjoyable activities (t(513) = 4.21, *p* < 0.001). The difference between the contributions of social support and enjoyable activities was not significant (t(513) = 1.75, *p* = 0.081). 

## 4. Discussion

The current study was designed to learn about the role of social support, self-compassion and self-care in predicting physical and mental health among mothers of young children. The main hypothesis of this study was that higher social support, self-compassion, and self-care would be associated with improved physical and mental health among mothers. The current study’s results, which align with this hypothesis, suggest that social support and self-compassion are pivotal factors in promoting physical and mental health among mothers of young children. Moreover, engagement in self-care activities is an essential element in promoting mothers’ mental health. 

It is well established that women caring for young children often face stress and physical challenges that can impact their overall mental and physical health. This, in turn, may affect the health and well-being of their children [33,34,35]. It seems that the variables examined in the current study better predicted the mothers’ mental health than their physical health. However, from a holistic perspective of the body and mind, physical health is known to affect mental health, and vice versa [36,37,38,39]. While caring for an infant and toddler in the early years can be demanding and intense, it is crucial to raise awareness of these issues and encourage their practice among postpartum women. 

Research indicates that social support can improve overall health, reduce emotional distress and loneliness, and improve mothers’ well-being and mental health outcomes [13]. Social support among postpartum women can promote health in various ways. Emotional support aids in coping with stress and difficulties, informative support offers vital information regarding the care of babies and toddlers, and instrumental support assists with everyday tasks such as cooking, laundry and accompanying women to clinics. The presence of supportive family members, friends and professionals provides a sense of security, relief from caregiving burdens, and an attentive other re feelings and concerns [40]. Thus, the potential of social support in enhancing women’s health is evident. However, it is crucial to avoid the creation of negative social support, which arises from providing help that the woman does not desire and may adversely affect her health [41]. This means that the provision of support should be done in accordance with the mother’s wishes and needs. 

Self-compassion means being supportive toward oneself when experiencing suffering or pain [15]. It may provide mothers with a constructive way to address any guilt they feel about prioritizing their own health [17], serving to promote positive well-being and mental health during the perinatal and early postnatal periods. Self-compassion also appears to be a powerful source of coping and resilience when faced with life stressors [20]. Meta-analysis reviews show that the inclusion of self-compassion components in parental interventions is positively correlated with improved parental self-compassion and lower levels of stress, depression and anxiety. Self-compassion was positively associated with coping with stress and adaptive behavior to life demands [42,43]. 

Another factor that can enhance women’s mental health and well-being is their engagement in enjoyable activities. Research shows that individuals who participate in more enjoyable leisure activities function better psychologically and physically and report better positive affect, life satisfaction, and more social support [22]. The respondents in the current study reported moderate-to-low participation in enjoyable activities and moderate self-compassion. It is important to note that respondents reporting higher social support also reported more participation in enjoyable activities and higher self-compassion, emphasizing the importance of having social support for mothers of young children. 

These findings demonstrate a combined need for both personal and social support to achieve positive health outcomes for mothers of young children. Social support is a crucial factor that helps these mothers, from a practical perspective, engage in self-care through enjoyable activities. However, self-compassion is also essential for them to prioritize these enjoyable activities, acknowledging that both the mother and her supportive environment view these as promoting activities rather than unnecessary indulgences. 

We further hypothesized that we would find differences in the mothers’ physical and mental health based on their socio-demographic characteristics (ethnicity, age, employment status and income level). Indeed, the results describe better physical and mental health reported among younger mothers and Jewish mothers, better mental health among working mothers, and better physical health among women reporting a higher socio-economic status. 

In Israel, the Arab community experiences significant health disparities compared to their Jewish counterparts. Arab women tend to have a higher prevalence of obesity, greater rates of depression, and more chronic health conditions than Jewish women. These differences may stem from various cultural, economic and/or social factors [44,45,46]. This study shows that Arab mothers face significant physical and mental health challenges. This finding is essential for developing initiatives to mitigate health disparities and improve mothers’ overall health and well-being. Integrating social support and self-compassion into such programs may be particularly beneficial for Arab mothers. This pattern may relate to increased negative social support in the Israeli Arab community, driven by different customs for new mothers. Arab culture involves frequent visits from family and community, offering help and acknowledgment through rituals, gifts, and meals. These traditions are less common in the Jewish community, and some Arab women might find them burdensome, possibly leading to higher negative social support and poorer health outcomes [47].

Our findings suggest that younger women tend to exhibit better physical and mental health. Although the advantages of better physical health at a younger age and quicker recovery after childbirth are clear, research shows that having a young maternal age at childbirth can pose risks for poor mental health after delivery. Research shows that older mothers tend to exhibit better physical and mental adjustment as compared to younger mothers [48], while Schmidt and her colleagues [49] emphasized the link between young maternal age at childbirth and higher rates of depression and anxiety. However, young age does not always have a direct or consistent impact on postpartum mental health. Young mothers who receive high levels of family support report better mental health as compared to mothers with limited support [50]. The impact of age may vary depending on the cultural and social context; at times, young mothers benefit from significant family support that reduces levels of distress, and at other times, the immaturity associated with young age poses a risk for lower mental health [49,50,51,52]. 

The findings of the multivariate analysis show that while women’s employment status was not found to predict their physical health, working mothers reported better mental health compared to non-working mothers. Work is a significant component of an individual’s sense of meaning, providing satisfaction, social connections, a support system and greater economic capacity. However, emphasis should always be placed on the central component of work intensity, since very high-intensity work, which may create home–family conflict, may have adverse effects on mothers’ mental health over time. A prior study that examined the biological health of postpartum women through telomere length indicated that full-time working mothers have shorter telomeres as compared to part-time or unemployed ones [53]. High levels of work stress combined with family responsibilities have been linked to an increased risk of mental health issues, particularly depression [54]. The current study involved mothers of children up to the age of three, a longer period after birth than in other studies. For this reason, the current findings may indicate that while early return to work after childbirth poses a risk factor for mothers’ mental and physical health [55], returning to work in the months and years after birth benefits their mental health and may even be a component of self-care and fulfillment.

The current study may be subject to several limitations. First, it was conducted in a cross-sectional design that does not allow for causal relationships. In addition, it was conducted among respondents using an online panel and, therefore, may be subject to self-selection bias and may not include populations with lower technological orientation. Finally, due to its quantitative nature, this study allows for limited elaboration on barriers and in-depth aspects related to self-care and self-compassion. 

## 5. Conclusions

Improved maternal health positively impacts children’s health and the well-being of the entire family. The current study emphasizes the significance of social support, self-compassion, and engaging in enjoyable activities, which are not merely leisure privileges but serve as a foundation for enhancing the physical and mental health of mothers with young children. 

Health and welfare practitioners, such as community nurses, social workers, psychologists, and others working with mothers of young children, should focus on promoting self-care and self-compassion interventions. Additionally, they should help these mothers create a nurturing environment, encourage social support, and enable them to practice self-care. 

The current findings can serve as a foundation for further research, including a mixed-methods approach that combines an in-depth cross-cultural qualitative study on self-compassion and self-care, and their expressions among mothers, alongside a quantitative study. Furthermore, an applied experimental intervention study is necessary to evaluate the effectiveness of strategies for promoting self-care and self-compassion.

## Figures and Tables

**Table 1:** Participants’ demographic and socioeconomic characteristics (N = 514).

Variable	Categories	Values
Age, M (SD), range		31.48 (5.64), 19–48
Marital status, n (%)	married, with a partner	475 (92.4)
	single, divorced	39 (7.6)
Number of children, M (SD), range		1.76 (1.08), 1–10
Time from last delivery, M (SD), range (months)		13.85 (9.31), 1–36
Time since end of maternity leave, n (%)	still on maternity leave	111 (21.6)
	a month to four months	100 (19.5)
	over four months	303 (58.9)
Level of education, n (%)	high school or professional training	163 (31.7)
	bachelor’s degree	220 (42.8)
	graduate degree	131 (25.5)
Employment status, n (%)	full-time	288 (56.0)
	part-time	105 (20.4)
	unemployed, on maternity leave, housewife	121 (23.6)
Income	below average	219 (42.6)
	average	144 (28.0)
	above average	151 (29.4)
Ethnicity, n (%)	Jewish	436 (84.8)
	Arab	78 (15.2)
Level of religiosity, n (%)	secular	304 (59.1)
	partly religious	120 (23.3)
	religious	90 (17.5)
Type of residence, n (%)	urban	425 (82.7)
	rural	89 (17.3)

**Table 2:** Means, standard deviations, and Pearson correlations for physical and mental health, social support, self-compassion, and enjoyable activities (N = 514).

	M (SD)	1. r (p)	2. r (p)	3. r (p)	4. r (p)	5. r (p)
1.Physical health	78.36 (21.00)	1				
2.Mental health	65.88 (20.28)	0.45 (*p* < 0.001)	1			
3.Social support	5.71 (1.22)	0.25 (*p* < 0.001)	0.46 (*p* < 0.001)	1		
4.Self-compassion	3.28 (0.70)	0.19 (*p* < 0.001)	0.52 (*p* < 0.001)	0.42 (*p* < 0.001)	1	
5.Enjoyable activities	15.82 (7.78)	0.12 (*p* = 0.005)	0.30 (*p* < 0.001)	0.28 (*p* < 0.001)	0.32 (*p* < 0.001)	1

Note. Range: physical and mental health 0–100, social support 1–7, self-compassion 1–5, enjoyable activities 0–40.

**Table 3:** Pearson and Spearman correlations and analyses of variance for physical and mental health by demographic and socio-economic variables (N = 514).

Demographic/Socio-Economic Variables		Physical Health		Mental Health	
		r (p)		r (p)	
Age		r_p_ = −0.06 (*p* = 0.143)		r_p_ = −0.10 (*p* = 0.021)	
Number of children at home		r_s_ = −0.03 (*p* = 0.478)		r_s_ = 0.02 (*p* = 0.625)	
Time from last baby delivery		r_p_ = −0.05 (*p* = 0.255)		r_p_ = −0.06 (*p* = 0.141)	
Income		r_s_ = 0.14 (*p* = 0.001)		r_s_ = 0.09 (*p* = 0.048)	
		**M (SD)**	**F(df)**	**M (SD)**	**F(df)**
Ethnicity	Jewish (n = 436)	M = 80.03 (SD = 19.89)	F(1, 511) = 18.93 (*p* < 0.001) (η^2^ = 0.036)	M = 67.18 (SD = 20.41)	F(1, 511) = 12.11 (*p* < 0.001) (η^2^ = 0.023)
	Arab (n = 78)	M = 68.99 (SD = 24.43)		M = 58.59 (SD = 18.03)	
Time since the end of maternity leave	still on maternity leave (n = 111)	M = 76.69 (SD = 23.06)	F(2, 511) = 1.30 (*p* = 0.274) (η^2^ = 0.005)	M = 63.97 (SD = 17.80)	F(2, 511) = 2.14 (*p* = 0.118) (η^2^ = 0.008)
	a month to four months (n = 100)	M = 81.19 (SD = 17.64)		M = 69.45 (SD = 19.07)	
	over four months (n = 303)	M = 78.03 (SD = 21.20)		M = 65.40 (SD = 21.41)	
Level of education	less than academic (n = 163)	M = 76.96 (SD = 20.78)	F(2, 511) = 0.55 (*p* = 0.578) (η^2^ = 0.002)	M = 67.23 (SD = 21.28)	F(2, 511) = 0.87 (*p* = 0.420) (η^2^ = 0.003)
	bachelor’s degree (n = 220)	M = 78.84 (SD = 21.59)		M = 65.93 (SD = 20.25)	
	graduate degree (n = 131)	M = 79.29 (SD = 20.31)		M = 64.10 (SD = 19.05)	
Employment status	unemployed, housewife, employed less than half of full-time job (n = 74)	M = 72.04 (SD = 26.38)	F(2, 511) = 3.97 (*p* = 0.019) (η^2^ = 0.015)	M = 55.42 (SD = 22.86)	F(2, 511) = 12.09 (*p* < 0.001) (η^2^ = 0.045)
	maternity leave (n = 61)	M = 78.89 (SD = 20.86)		M = 66.56 (SD = 18.30)	
	full time, part time above half of full-time job (n = 379)	M = 79.50 (SD = 19.64)		M = 67.81 (SD = 19.47)	
Level of religiosity	secular (n = 304)	M = 79.19 (SD = 20.46)	F(2, 511) = 0.76 (*p* = 0.466) (η^2^ = 0.003)	M = 64.77 (SD = 20.44)	F(2, 511) = 2.64 (*p* = 0.072) (η^2^ = 0.010)
	partly religious (n = 120)	M = 76.41 (SD = 21.97)		M = 65.38 (SD = 19.49)	
	religious (n = 90)	M = 78.12 (SD = 21.51)		M = 70.29 (SD = 20.43)	

Note. The Bonferroni correction for multiple comparisons was applied per dependent variable (*p* < 0.005). For number of children at home and income: Spearman correlations. For age and time since last baby delivery: Pearson correlations.

**Table 4:** Multiple regression models for physical and mental health with basic demographic and socio-economic variables, social support, self-compassion, and enjoyable activities (N = 514).

	Physical Health			Mental Health		
	B (SE)	β	*p*	B (SE)	β	*p*
Step 1						
Ethnicity (Jewish)	9.68 (2.61)	0.17	<0.001	8.35 (2.51)	0.15	<0.001
Age	−0.49 (0.17)	−0.13	0.004	−0.49 (0.16)	−0.14	0.003
Employment status (employed)	4.36 (2.67)	0.07	0.104	10.97 (2.57)	0.19	<0.001
Income (high)	2.31 (0.83)	0.14	0.006	0.61 (0.80)	0.04	0.448
Adj R^2^	0.06, *p* < 0.001			0.07, *p* < 0.001		
Step 2						
Ethnicity (Jewish)	9.59 (2.62)	0.16	<0.001	8.57 (2.10)	0.15	<0.001
Age	−0.43 (0.17)	−0.12	0.010	−0.37 (0.13)	−0.10	0.006
Employment status (employed)	1.94 (2.64)	0.03	0.463	5.65 (2.12)	0.10	0.008
Income (high)	2.08 (0.81)	0.12	0.011	0.28 (0.66)	0.02	0.666
Social support	2.30 (0.83)	0.13	0.006	3.79 (0.67)	0.23	<0.001
Self-compassion	3.45 (1.42)	0.12	0.015	10.88 (1.14)	0.38	<0.001
Enjoyable activities	0.17 (0.12)	0.06	0.180	0.31 (0.10)	0.12	0.002
Adj R^2^	0.11, *p* < 0.001			0.39, *p* < 0.001		
F(7, 506)	10.32, *p* < 0.001			46.94, *p* < 0.001		

## Data Availability

The data presented in this study are available on request from the corresponding author. The data are not publicly available to preserve the respondents’ privacy.
